# Factors Predicting Delay of Medical and Dental Care Among American Adults Over 50 During the COVID-19 Pandemic

**DOI:** 10.1093/geroni/igab046.1773

**Published:** 2021-12-17

**Authors:** Athena Chung Yin Chan, Rodlescia Sneed

**Affiliations:** 1 University of Minnesota -- Twin Cities, St Paul, Minnesota, United States; 2 Michigan State University, Flint, Michigan, United States

## Abstract

Delaying or avoiding medical care is associated with a range of poor health-related outcomes. Due to concerns about the coronavirus (COVID) pandemic, many older adults delayed or avoided seeking medical and dental care in 2020. The purpose of this study was to examine factors predicting delay or avoidance of care among older adults. Participants were U.S. adults aged ≥ 50 who participated in the Health and Retirement Study, a population-based study of community-dwelling adults. Delays in seeking or obtaining medical and dental care were assessed via self-report in June 2020. Hierarchical logistic regression models were used to predict the influence of demographic variables (e.g. age, marital status, race and ethnicity, gender, education, work status), health insurance status, health status, and COVID-related experiences (self or household history of COVID diagnosis, knowing anyone who died from COVID, willingness to take risks, and pandemic concerns) on delay or avoidance of medical or dental care. Overall, 30% of our sample reported delaying or avoiding medical or dental care. Delayed care was lower among younger age (0.97; 0.96-0.99); non-Hispanic Blacks (0.59; 0.43-0.80), Hispanics (0.63; 0.45-0.87) and women (0.78; 0.63-0.97). Moreover, care avoidance was significantly higher among persons with disability or on sick leave 1.56; 1.04-2.33), those with chronic diseases (1.41; 1.00-2.01), those with fair/poor self-rated health (1.33; 1.03-1.73), and those with high COVID-related concerns (1.34; 1.07-1.68). Understanding factors associated with medical care will inform targeted care delivery and health promotion encouraging persons in need to safely seek timely healthcare services.

